# Tumor blood flow and apparent diffusion coefficient histogram analysis for differentiating malignant salivary tumors from pleomorphic adenomas and Warthin’s tumors

**DOI:** 10.1038/s41598-022-09968-2

**Published:** 2022-04-08

**Authors:** Fumine Tanaka, Maki Umino, Masayuki Maeda, Ryohei Nakayama, Katsuhiro Inoue, Ryota Kogue, Makoto Obara, Hajime Sakuma

**Affiliations:** 1 Department of Radiology, Mie University School of Medicine, 2-174 Edobashi, Tsu, Mie Japan; 2 Department of Neuroradiology, Mie University School of Medicine, 2-174 Edobashi, Tsu, Mie Japan; 3 Department of Electronic and Computer Engineering, Ritsumeikan University, 1-1-1 Noji-higashi, Kusatsu, Shiga Japan; 4 Department of Radiology, Mie University Hospital, 2-174 Edobashi, Tsu, Mie Japan; 5 MR Clinical Science, Philips Japan, 2-13-37 Konan, Minato, Tokyo, Japan

**Keywords:** Cancer, Oncology

## Abstract

We aimed to assess the combined diagnostic value of apparent diffusion coefficient (ADC) and tumor blood flow (TBF) obtained by pseudocontinuous arterial spin labeling (pCASL) for differentiating malignant tumors (MTs) in salivary glands from pleomorphic adenomas (PAs) and Warthin’s tumors (WTs). We used pCASL imaging and ADC map to evaluate 65 patients, including 16 with MT, 30 with PA, and 19 with WT. We evaluated all tumors by histogram analyses and compared various characteristics by one-way analysis of variance followed by Tukey post-hoc tests. Diagnostic performance was evaluated by receiver operating characteristic (ROC) curve analysis. There were significant differences in the mean, 50th, 75th, and 90th percentiles of TBF among the tumor types, in the mean TBFs (mL/100 g/min) between MTs (57.47 ± 35.14) and PAs (29.88 ± 22.53, *p* = 0.039) and between MTs and WTs (119.31 ± 50.11, *p* < 0.001), as well as in the mean ADCs (× 10^−3^ mm^2^/s) between MTs (1.08 ± 0.28) and PAs (1.60 ± 0.34, *p* < 0.001), but not in the mean ADCs between MTs and WTs (0.87 ± 0.23, *p* = 0.117). In the ROC curve analysis, the highest areas under the curves (AUCs) were achieved by the 10th and 25th percentiles of ADC (AUC = 0.885) for differentiating MTs from PAs and the 50th percentile of TBF (AUC = 0.855) for differentiating MTs from WTs. The AUCs of TBF, ADC, and combination of TBF and ADC were 0.850, 0.885, and 0.950 for MTs and PAs differentiation and 0.855, 0.814, and 0.905 for MTs and WTs differentiation, respectively. The combination of TBF and ADC evaluated by histogram analysis may help differentiate salivary gland MTs from PAs and WTs.

## Introduction

Parotid tumors represent nearly 70% of all salivary gland tumors, and 80% of them are benign^1^. The most frequent benign salivary gland tumors are pleomorphic adenomas (PAs), which comprise 45% of all salivary gland tumors, followed by Warthin’s tumors (WTs)^2^. On the other hand, malignant tumors (MTs) represent nearly 20% of parotid tumors, approximately 40% of submandibular tumors, and 70–90% of sublingual tumors^1,3^.

Malignant salivary tumors demonstrate a range of biological behaviors. About 40% of MTs are indolent, especially in young adults^3^. The other 40% of MTs are aggressive, especially in the elderly^3^. Clinical indicators suggesting MTs are rapid growth rate, pain, facial nerve involvement, and cervical adenopathy. However, a slow growth rate of asymptomatic mass does not exclude MTs^3^. Therefore, it is important to differentiate MTs from benign salivary gland tumors, such as PAs and WTs. Fine-needle aspiration cytology is widely accepted as a reliable way to diagnose salivary gland tumors before surgical resection, but it is not appropriate for tumors located in deep areas and is an intrinsically invasive procedure^4^. Noninvasive magnetic resonance imaging (MRI) techniques may improve the diagnostic performance of salivary gland tumors regardless of tumor locations. However, conventional MRI cannot clearly distinguish between benign and malignant salivary gland tumors^5^. For instance, the apparent diffusion coefficient (ADC) obtained by diffusion-weighted imaging (DWI) reportedly provided useful information for the differentiation of WTs and PAs but remained inconclusive for differentiation of benign and malignant salivary gland tumors^6–8^.

Recently, arterial spin labeling (ASL) techniques, such as pulsed ASL or pseudocontinuous ASL (pCASL), were introduced for clinical applications^9^. This method has been applied for noninvasive measurement of tumor blood flow (TBF) by using the magnetization of protons in arterial blood as an intrinsic tracer without an exogenous contrast agent^9^. There have been only a few reports on the usefulness of ASL for differentiating salivary gland tumors so far^10–12^. The use of multiparametric MRI, such as DWI and ASL, may help radiologists by increasing their efficiency in the differential diagnosis of salivary gland tumors. This is because this method may decrease unnecessary examinations and invasive procedures, such as biopsies. We aimed to assess the combined diagnostic value of ADC and TBF for differentiating MTs in salivary glands from PAs and WTs.

## Results

A total of 65 subjects (age range, 11–86 years; mean 59 years; 34 males and 31 females) were finally included. There were 16 subjects with MTs, 30 with PAs, and 19 with WTs. The characteristics of patients are described in Table 1. The pathology of MTs was variable, including five carcinoma ex pleomorphic adenomas, two acinic-cell carcinomas, two adenocarcinomas, two adenoid cystic carcinomas, two mucoepidermoid carcinomas, one basal-cell adenocarcinoma, one epithelial myoepithelial carcinoma, and one salivary-duct carcinoma. One patient with PAs and eight patients with WTs had multiple or bilateral tumors. Among these patients, only the largest one was assessed.

**Table 1:** Patients’ characteristics.

	MT (n = 16)	PA (n = 30)	WT (n = 19)	*P* value
**Sex**				0.001*
Male:Female	9:7	9:21	16:3	
**Age**				0.002*
Range (year)	11–82	24–86	56–83	
Mean age (year)	60	53	68	
**Tumor diameter**				0.092
Range (mm)	14–96	11–60	17–64	
Mean (mm)	34.63	27.37	36.37	
**Tumor sub-site**				0.052
Parotid gland	11	22	19	
Submandibular gland	4	8	0	
Sublingual gland	1	0	0	
**Diagnostic method**				0.410
Resection	14	23	13	
Fine-needle aspiration cytology	2	7	6	

*MT* malignant tumor, *PA* pleomorphic adenoma, *WT* Warthin’s tumor.

*P value < 0.05.

### Comparison of the parameters for TBF and ADC between MTs, PAs, and WTs

**Figure 1:**
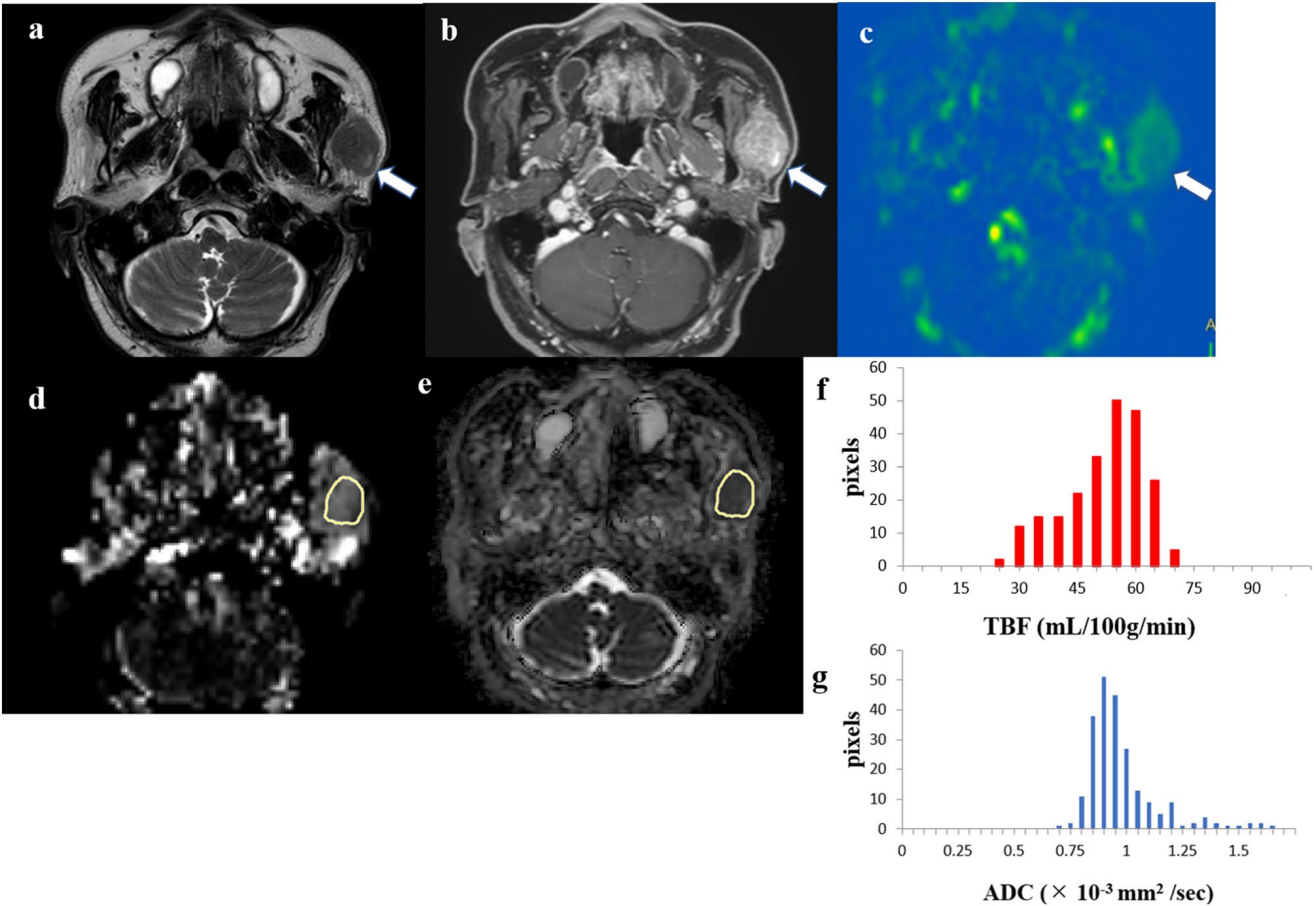
An 80-year-old male patient with a carcinoma ex pleomorphic adenoma in the left parotid gland. T2-weighted image (**a**) showing an iso signal intensity lesion (*arrow*). Contrast-enhanced 3D-T1-weighted image (**b**) showing homogeneous contrast enhancement (*arrow*). Tumor blood flow (TBF) color map (**c**) showing medium TBF (*arrow*). The region of interest (ROI) was manually drawn on the apparent diffusion coefficient (ADC) map of the software (**e**, *yellow*), and the ROI was copied from the ADC map to the TBF map of the software (**d**, *yellow*). The TBF histogram (**f**) and ADC histogram (**g**) are presented. The 50th percentile of the TBF value was 50.92 mL/100 g/min, whereas the 10th percentile of the ADC value was 0.82 × 10^−3^ mm^2^/s.

**Figure 2:**
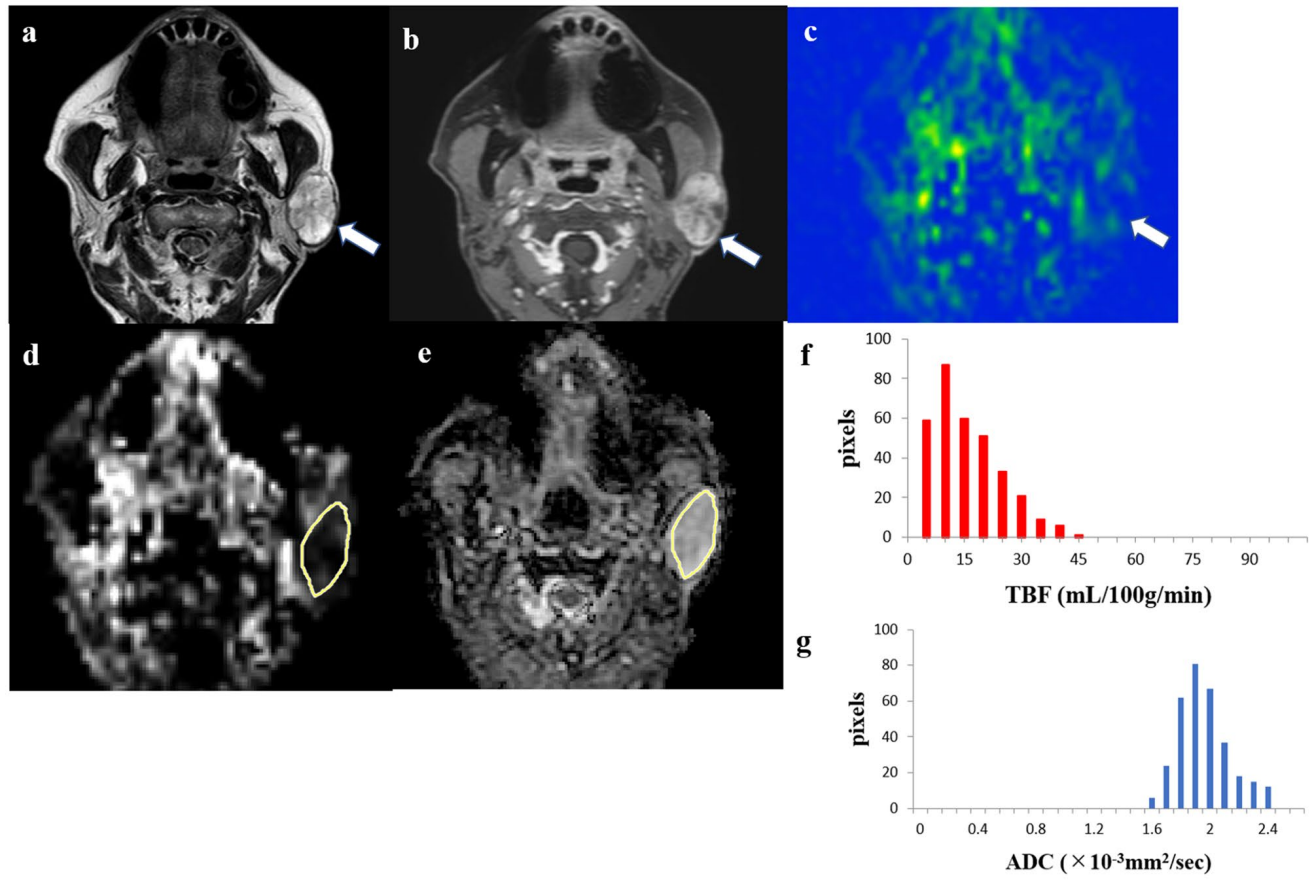
A 77-year-old female patient with a pleomorphic adenoma in the left parotid gland. T2-weighted image (**a**) showing a high signal intensity lesion (*arrow*). Contrast-enhanced 3D-T1-weighted image (**b**) showing a little heterogeneous contrast enhancement (*arrow*). Tumor blood flow (TBF) color map (**c**) showing low TBF (*arrow*). The region of interest (ROI) was manually drawn on the apparent diffusion coefficient (ADC) map of the software (**e**, *yellow*), and the ROI was copied from the ADC map to the TBF map of the software (**d**, *yellow*). The TBF histogram (**f**) and ADC histogram (**g**) are presented. The 50th percentile of the TBF value was 11.17 mL/100 g/min, whereas the 10th percentile of the ADC value was 1.71 × 10^−3^ mm^2^/s.

**Figure 3:**
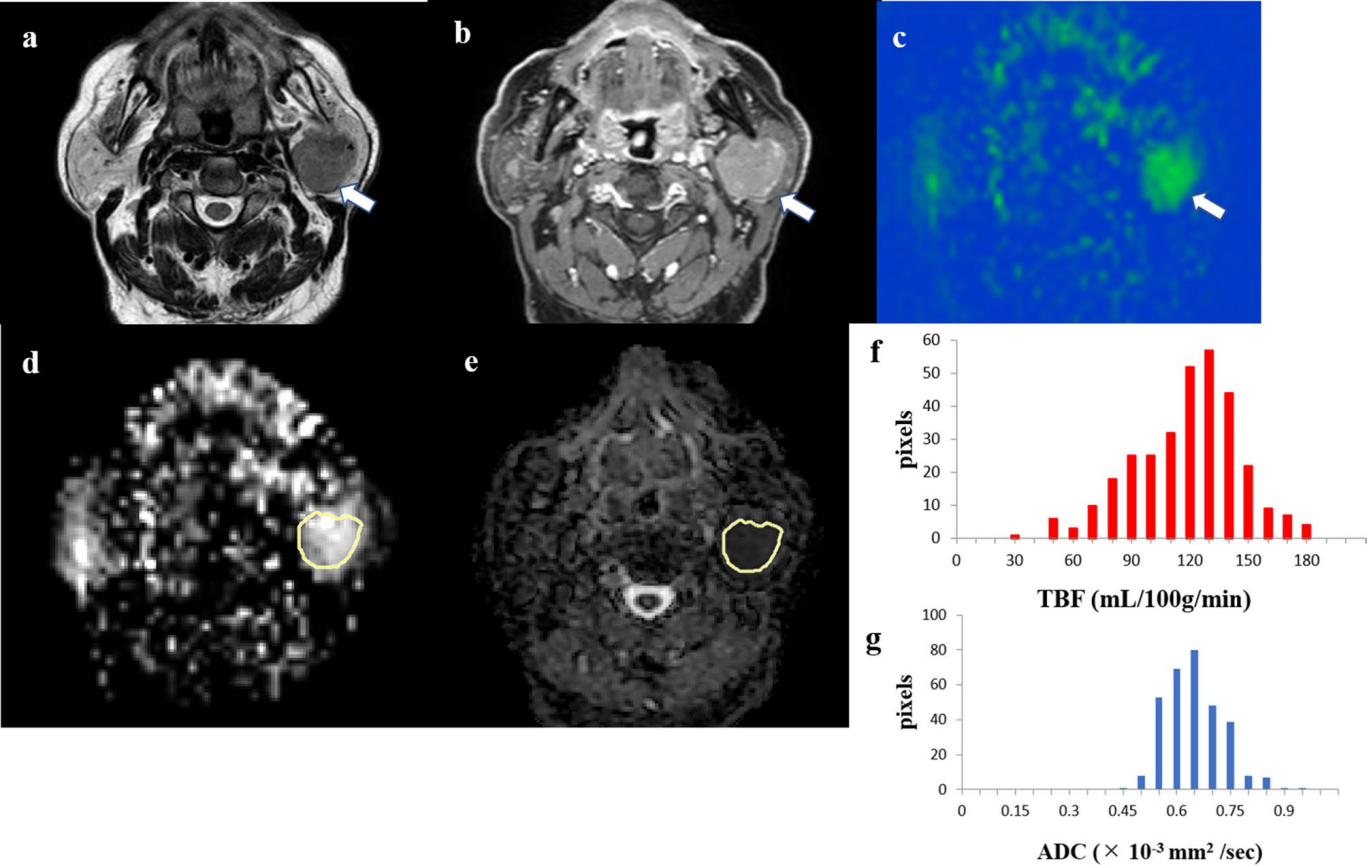
An 83-year-old male patient with a Warthin’s tumor in the left parotid gland. T2-weighted image (**a**) showing iso signal intensity lesion (*arrow*). Contrast-enhanced 3D-T1-weighted image (**b**) showing homogeneous contrast enhancement (*arrow*). Tumor blood flow (TBF) color map (**c**) showing high TBF (*arrow*). The region of interest (ROI) was manually drawn on the apparent diffusion coefficient (ADC) map of the software (**e**, *yellow*), and the ROI was copied from the ADC map to the TBF map of the software (**d**, *yellow*). TBF histogram (**f**) and ADC histogram (**g**) are presented. The 50th percentile of the TBF value was 117.90 mL/100 g/min, whereas the 10th percentile of ADC value was 0.53 × 10^−3^ mm^2^/s.

Figures 1, 2, and 3 show representative cases of MTs, PAs, and WTs, respectively. Supplementary Table S1 shows the results of Shapiro–Wilk test for each parameter. Tables 2 and 3 show the parameter measurements of TBF and ADC, respectively, in MTs, PAs, and WTs.

**Table 2:** Measurements of TBF in MTs, PAs, and WTs.

TBF parameters	Mean ± standard deviation			*P* value		
	MT	PA	WT	MT versus PA	MT versus WT	PA versus WT
Max	97.70 ± 54.98	66.11 ± 29.27	166.03 ± 49.85	0.054	< 0.001*	< 0.001*
Min	22.47 ± 29.10	7.22 ± 14.45	63.74 ± 43.62	0.219	< 0.001*	< 0.001*
Mean	57.47 ± 35.14	29.88 ± 22.53	119.31 ± 50.11	0.039*	< 0.001*	< 0.001*
10th percentile	34.37 ± 30.95	14.19 ± 18.84	88.96 ± 48.85	0.127	< 0.001*	< 0.001*
25th percentile	43.99 ± 32.73	20.84 ± 21.80	103.44 ± 50.52	0.09	< 0.001*	< 0.001*
50th percentile	56.36 ± 35.35	28.48 ± 23.62	120.80 ± 51.76	0.044*	< 0.001*	< 0.001*
75th percentile	70.71 ± 40.83	37.65 ± 25.32	135.35 ± 52.47	0.021*	< 0.001*	< 0.001*
90th percentile	81.82 ± 46.63	47.29 ± 25.87	147.45 ± 51.63	0.020*	< 0.001*	< 0.001*
Skewness	0.10 ± 0.52	0.64 ± 0.68	− 0.23 ± 0.72	0.029*	0.289	< 0.001*
Kurtosis	− 0.42 ± 0.49	0.62 ± 1.15	0.39 ± 2.82	0.136	0.364	0.887

*TBF* tumor blood flow (mL/100 g/min),* max* maximum, *min* minimum, *MT* malignant tumor,* PA* pleomorphic adenoma, *WT* Warthin's tumor.

*P value < 0.05.

**Table 3:** Measurements of ADC in MTs, PAs, and WTs.

ADC parameters	Mean ± standard deviation			*P* value		
	MT	PA	WT	MT versus PA	MT versus WT	PA versus WT
Max	1.39 ± 0.31	1.95 ± 0.37	1.29 ± 0.42	< 0.001*	0.684	< 0.001*
Min	0.76 ± 0.23	1.23 ± 0.38	0.51 ± 0.16	< 0.001*	0.050	< 0.001*
Mean	1.08 ± 0.28	1.60 ± 0.34	0.87 ± 0.23	< 0.001*	0.117	< 0.001*
10th percentile	0.90 ± 0.23	1.40 ± 0.33	0.68 ± 0.15	< 0.001*	0.052	< 0.001*
25th percentile	0.98 ± 0.27	1.49 ± 0.33	0.76 ± 0.17	< 0.001*	0.057	< 0.001*
50th percentile	1.08 ± 0.31	1.59 ± 0.34	0.85 ± 0.22	< 0.001*	0.075	< 0.001*
75th percentile	1.16 ± 0.31	1.71 ± 0.36	0.97 ± 0.30	< 0.001*	0.220	< 0.001*
90th percentile	1.25 ± 0.30	1.80 ± 0.37	1.09 ± 0.39	< 0.001*	0.393	< 0.001*
Skewness	− 0.03 ± 0.64	0.07 ± 0.47	0.29 ± 0.46	0.805	0.168	0.32
Kurtosis	0.29 ± 1.16	− 0.04 ± 0.95	0.55 ± 1.15	0.594	0.749	0.155

*ADC* apparent diffusion coefficient (× 10^−3^ mm^2^/s), *max* maximum, *min* minimum, *MT* malignant tumor, *PA* pleomorphic adenoma, *WT* Warthin’s tumor.

*P value < 0.05.

There were significant differences in the mean, 50th, 75th, and 90th percentiles of TBF among all three types of tumors (all *p* < 0.05). The mean TBF was significantly higher in MTs (57.47 ± 35.14 mL/100 g/min) than in PAs (29.88 ± 22.53 mL/100 g/min, *p* = 0.039) and significantly lower in MTs than in WTs (119.31 ± 50.11 mL/100 g/min, *p* < 0.001). The 50th percentile of TBF was significantly higher in MTs (56.36 ± 35.35 mL/100 g/min) than in PAs (28.48 ± 23.62 mL/100 g/min, *p* = 0.044) and significantly lower in MTs than in WTs (120.80 ± 51.76 mL/100 g/min, *p* < 0.001). The 75th percentile of TBF was significantly higher in MTs (70.71 ± 40.83 mL/100 g/min) than in PAs (37.65 ± 25.32 mL/100 g/min, *p* = 0.021) and significantly lower in MTs than in WTs (135.35 ± 52.47 mL/100 g/min, *p* < 0.001). The 90th percentile of TBF was significantly higher in MTs (81.82 ± 46.63 mL/100 g/min) than in PAs (47.29 ± 25.87 mL/100 g/min, *p* = 0.020) and significantly lower in MTs than in WTs (147.45 ± 51.63 mL/100 g/min, *p* < 0.001).

There was a significant difference in the mean ADCs between MTs (1.08 ± 0.28 × 10^−3^ mm^2^/s) and PAs (1.60 ± 0.34 × 10^−3^ mm^2^/s, *p* < 0.001) but not between MTs and WTs (0.87 ± 0.23 × 10^−3^ mm^2^/s, *p* = 0.117). There were no ADC parameters that showed significant differences for all three combinations of tumor types (MT and PA, MT and WT, and PA and WT).

### Comparison of diagnostic performance for TBF and ADC in differentiating MTs, PAs, and WTs

Supplementary Tables S2, S3, and S4 show the diagnostic performance of each parameter determined by the receiver operating characteristic (ROC) curve analysis. When differentiating MTs from PAs, the 10th and 25th percentiles of the ADC both had the best diagnostic performance out of all TBF and ADC parameters, with areas under the curve (AUCs) of 0.885 (95% confidence interval [CI], 0.787–0.984, *p* < 0.001) and 0.885 (95% CI, 0.787–0.984, *p* < 0.001), respectively, which is considered medium diagnostic performance. The best detected cutoff points were 1.15 × 10^−3^ mm^2^/s and 1.26 × 10^−3^ mm^2^/s, respectively, yielding sensitivity and specificity for both cutoff values of 73.3% and 93.8%, respectively.

When differentiating MTs from WTs, the 50th percentile of TBF had the best diagnostic performance out of all TBF and ADC, with an AUC of 0.855 (95% CI, 0.733–0.977, *p* < 0.001), which is considered medium diagnostic performance. The best detected cutoff point was 78.02 mL/100 g/min, yielding a sensitivity and a specificity of 84.2% and 75.0%, respectively.

When differentiating PAs from WTs, the 10th percentile of ADC had the best diagnostic performance out of all TBFs and ADCs, with an AUC of 0.984 (95% CI, 0.958–1.000, *p* < 0.001), which is considered high diagnostic performance. The best detected cutoff point was 0.79 × 10^−3^ mm^2^/s, yielding a sensitivity and a specificity of 100.0% and 89.5%, respectively.

**Figure 4:**
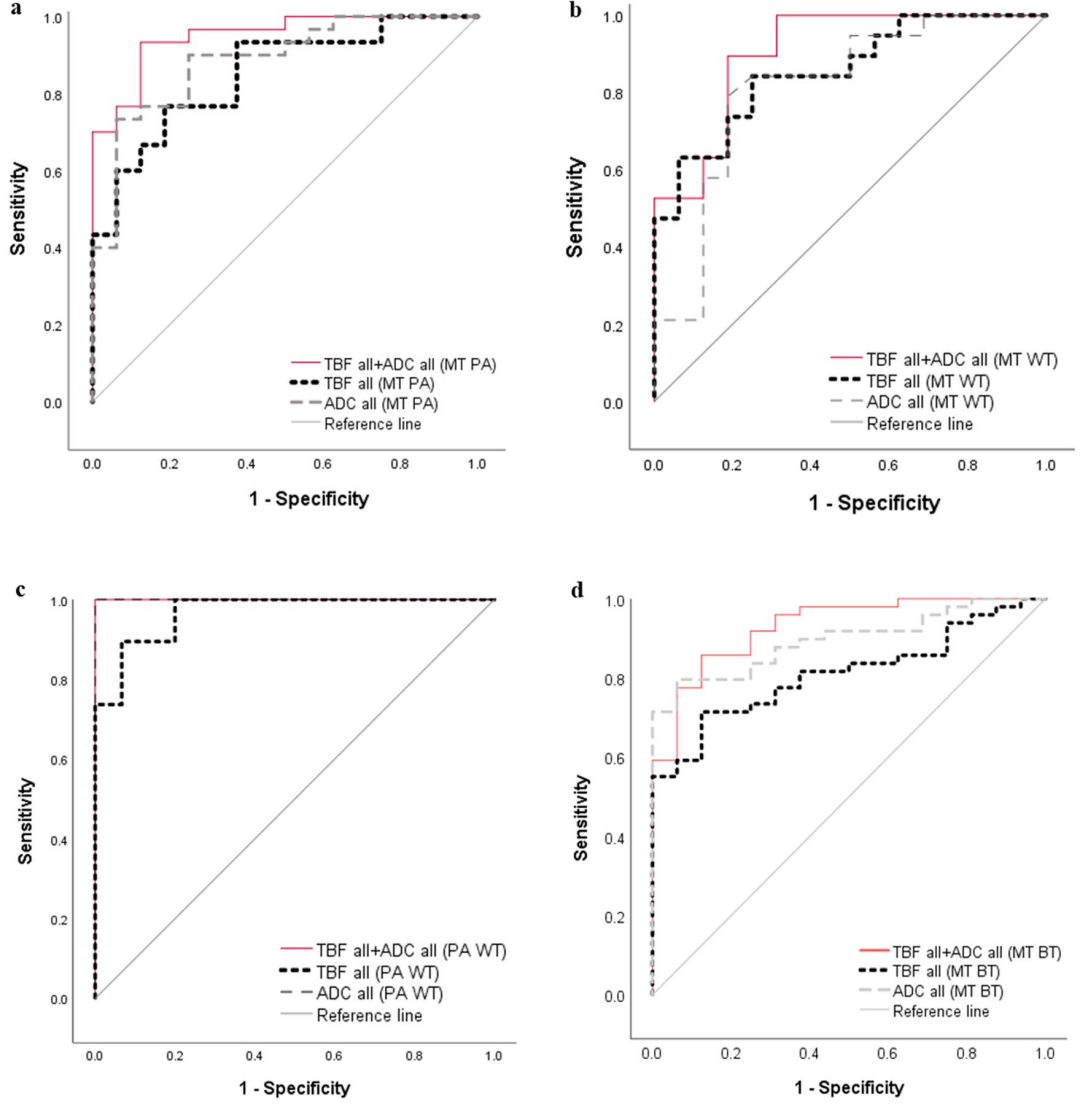
ROC curve analyses for differentiating malignant tumors (MT) from pleomorphic adenomas (PA) (**a**), for differentiating MT from Warthin’s tumors (WT) (**b**), for differentiating PA from WT (**c**), and for differentiating MT from benign tumors (BT), including PA and WT (**d**). (**a**) The areas under the curve (AUCs) for all tumor blood flow parameters (TBF_all_) and all apparent diffusion coefficients (ADC_all_) indicate medium diagnostic performances for both (AUC = 0.850 and 0.885, respectively). Conversely, the AUC for the combination of the TBF_all_ and ADC_all_ indicates high diagnostic performance (AUC = 0.950). (**b**) The AUCs for the TBF_all_ and ADC_all_ indicate medium diagnostic performances for both (AUC = 0.855 and 0.814, respectively), whereas the AUC for the combination of TBF_all_ and ADC_all_ indicates high diagnostic performance (AUC = 0.905). (**c**) The AUCs of the TBF_all_, ADC_all_, and combination of TBF_all_ and ADC_all_ all indicate high diagnostic performances (AUC = 0.968, 1.000, and 1.000, respectively). (**d**) The AUCs for the TBF_all_ and ADC_all_ indicate medium diagnostic performances for both (AUC = 0.811 and 0.895, respectively), whereas the AUC for the combination of TBF_all_ and ADC_all_ indicates high diagnostic performance (AUC = 0.930).

Figure 4 summarizes the diagnostic performance of the parameters. In differentiating MTs from PAs, the AUC for the combination of TBF_all_ and ADC_all_ (0.950; 95% CI, 0.892–1.000, *p* < 0.001) was higher than those for TBF_all_ alone (0.850; 95% CI, 0.739–0.961, *p* < 0.001) and ADC_all_ alone (0.885; 95% CI, 0.787–0.984, *p* < 0.001), which suggests that the diagnostic performance improved from medium to high with the combination of TBF_all_ and ADC_all_. In differentiating MTs from WTs, the AUC for the combination of TBF_all_ and ADC_all_ (0.905; 95% CI, 0.805–1.000, *p* < 0.001) was higher than those for TBF_all_ alone (0.855; 95% CI, 0.733–0.977, *p* < 0.001) and ADC_all_ alone (0.814; 95% CI, 0.664–0.964, *p* = 0.002), which suggests that the diagnostic performance improved from medium to high with the combination of TBF_all_ and ADC_all_. In differentiating PAs from WTs, the AUC for the combination of TBF_all_ and ADC_all_ (1.000; 95% CI, 1.000–1.000, *p* < 0.001) was higher than that for TBF_all_ alone (0.968; 95% CI, 0.929–1.000, *p* < 0.001) and the same as that for ADC_all_ alone (1.000; 95% CI, 1.000–1.000, *p* < 0.001), which suggested a medium diagnostic performance for TBF_all_ alone and high performance for both ADC_all_ alone and the combination of TBF_all_ and ADC_all_. In differentiating MTs from benign tumors (BTs), including PAs and WTs, the AUC for the combination of TBF_all_ and ADC_all_ (0.930; 95% CI, 0.865–0.995, *p* < 0.001) was higher than those for TBF_all_ alone (0.811; 95% CI, 0.709–0.914, *p* < 0.001) and ADC alone (0.895; 95% CI, 0.821–0.970, *p* < 0.001), which suggests that the diagnostic performance improved from medium to high with the combination of TBF_all_ and ADC_all_.

Supplementary Figure S1 presents the scatter plots for MTs, PAs, and WTs, which represent the propensity scores of TBF_all_ and ADC_all_ for each tumor.

### Interobserver agreement of TBF and ADC measurements

Supplementary Table S5 shows the intraclass correlation coefficients (ICCs) of the measurements by the two observers. Excellent agreements were observed for all parameters except for the skewness of ADC, which showed good agreement.

## Discussion

In this study, the diagnostic performance of the combination of TBF_all_ and ADC_all_ for differentiating MTs from PAs and WTs improved relative to the performance of each parameter alone. However, in differentiating PAs from WTs, the diagnostic performance of ADC_all_ alone showed perfect discrimination, and therefore, the value of adding the combination of ADC_all_ and TBF_all_ was low. To our best knowledge, this is the first study to evaluate the usefulness of the combination of pCASL and the ADC map by histogram analysis for differentiating malignant salivary gland tumors from PAs and WTs.

According to Kato et al., qualitative analysis showed that TBF was significantly higher in WTs than PAs and MTs but did not show a significant difference between PAs and MTs^10^. However, we demonstrated that the mean, 50th, 75th, and 90th percentiles of TBF could differentiate MTs, PAs, and WTs. We speculate that the differences in ASL methods may explain why their results differed from ours. They placed the regions of interest (ROIs) on both a tumor and the contralateral normal parotid gland parenchyma at the same level and then evaluated tumor-to-parotid signal intensity ratios from ASL images supposing that those ratios are surrogates of TBF^10^. They measured the relative ratio of salivary gland tumors to normal parotid glands, whereas we measured the TBF values of tumors quantitatively. Consequently, histogram analysis may overcome the limitations of qualitative analysis. Moreover, they used an alternating radio-frequency ASL sequence with gradient echo-type single-shot echo-planar imaging (MP-EPISTAR), which suffers from susceptibility artifacts more seriously than pCASL sequences that use 3D turbo spin-echo (TSE) acquisition^10^. In addition, MP-EPISTAR used in the study of Kato et al. has a lower signal-to-noise ratio than that of pCASL^11^. Thus, the pCASL technique may be more suitable for imaging compared to the ASL sequence that Kato et al. used for differentiation among MTs, PAs, and WTs.

A recent report stated that metrics, such as percentiles, kurtosis, and skewness, calculated by histogram analysis are strong and reliable quantitative surrogate markers of tumor heterogeneity^13^. Thus, we consider that microenvironments of tumors could be masked by evaluating only a single parameter, such as the mean value. Yamamoto et al. demonstrated that the mean TBF value was significantly higher in WTs than in PAs by using the pCASL sequence with conventional ROI analysis^11^. They also showed that the higher mean TBF of WTs than of PAs was attributable to higher micro-vessel density in WTs than in PAs^11^. Furthermore, our results revealed that the 75th and 90th percentiles of TBF exhibited higher AUC values than the mean TBF. Consequently, histogram analysis appears to provide more detailed information about TBF.

Kato et al. reported that the mean ADC values were significantly higher in PAs than in WTs and MTs but were not significantly different between WTs and MTs^10^. Their results were consistent with our results showing that all ADC parameters except for skewness and kurtosis were significantly different between PAs and WTs and between PAs and MTs, but not between WTs and MTs. Razek et al. studied ADC values by histogram analysis for diagnosis of PAs, WTs, and MTs and reported significant differences in the means and skewness of ADC among all three tumors, although these differences between WTs and MTs were weaker than those between PAs and WTs and PAs and MTs^14^. Histopathologically, PAs comprise an abundant myxoma-like stroma^6,11^, which probably contributed to the highest value obtained for it among the three types of tumors in all ADC parameters, except for the skewness and kurtosis values for ADC, in our study. In contrast, WTs showed the lowest value among all ADC parameters, except for the skewness and kurtosis values for ADC, which might reflect epithelial and lymphoid stromata with microscopic slit-like cysts filled with proteinous fluid^2,6^.

Regarding the other conventional method, time-intensity curve patterns on dynamic contrast-enhanced MRI were found useful in the differentiation of salivary gland tumors^15^. Nevertheless**,** it requires contrast media, which can be harmful to patients with renal dysfunction or allergies to these materials. Moreover, dynamic contrast-enhanced MRI only allows for one series of scans. In contrast, ASL can overcome these drawbacks and allows for repeat scanning without any contrast agents. Further, the time-intensity curve cannot provide quantitative data. For that reason, we focused on the noninvasive and quantitative MRI techniques of pCASL and ADC.

There were several limitations in this study. First, the study was conducted at a single institution with a relatively small number of subjects. Further studies with a larger number of subjects and a wider range of benign and malignant tumor types are required to confirm the efficacy of pCASL imaging and ADC map in evaluating salivary gland tumors. Furthermore, we should consider classifying malignant tumors into low, intermediate, and high grades and evaluate each group to facilitate the management of patients at an earlier stage. Regarding the analytical method, we could not evaluate the whole pCASL image slices and ADC maps for each tumor. Particularly, MTs tend to have heterogenous characteristics. Thus, whole-tumor evaluation would be desirable in future studies. Moreover, we evaluated limited parameters in histogram analysis. Thus, we need to consider other parameters, such as entropy, to provide further information on tumor heterogeneity.

In conclusion, the combination of TBF and ADC evaluated by histogram analysis was found to be helpful for differentiating MTs from PAs and WTs in salivary glands.

## Methods

### Subjects

This study was approved by the ethics committee of our university, and the requirement for written informed consent was waived because of the retrospective study design. All procedures were conducted according to the principles of the World Medical Association Declaration of Helsinki. We retrospectively identified 170 patients suspected of salivary gland tumors who had undergone pretreatment MRI between December 2015 and September 2020. Patients who fulfilled the following criteria were included: (a) available preoperative 3 T MRI with sufficient image quality, including pCASL images, DWI, T1-weighted images, contrast-enhanced T1-weighted images, and T2-weighted images; (b) tumor size > 10 mm; (c) tumors pathologically proven using fine-needle aspiration biopsy or surgical resection; and (d) diagnosis of MT, PA, or WT of the salivary gland.

Patients were excluded on the absence of definitive diagnosis from biopsy or surgical resection (n = 37); histological diagnosis other than MT, PA, or WT (n = 19); lack of contrast-enhanced T1-weighted images (n = 20); lesions with large necrosis, cysts, hemorrhage, or infectious complications (n = 11); tumors smaller than 10 mm (n = 2); ADC map with artifact (n = 1); patients using a different pCASL protocol (n = 3); and data loading error in software (n = 12). A total of 65 patients met our inclusion criteria.

### Conventional MRI protocol

All patients underwent MRI on a 3 T MRI system (Ingenia; Philips Medical Systems, Best, the Netherlands) with a Head/Neck coil. The pulse sequence parameters were as follows. T2-weighted imaging with a TSE sequence: repetition time (TR)/echo time (TE), 6528/90 ms; number of signals averaged (NSA), 1; field of view (FOV), 240 × 240 mm; matrix, 384 × 271; slice thickness, 4 mm; number of slices, 22; acceleration factor, 1.5; and scanning time, 1 min 57 s. T1-weighted imaging with a TSE sequence: TR/TE, 614/14 ms; NSA, 1; FOV, 240 × 240 mm; matrix, 352 × 246; slice thickness, 4 mm; number of slices, 22; acceleration factor, 2; and acquisition time, 2 min 34 s. DWI with a spin-echo, echo-planar sequence: TR/TE, 5000/88 ms; fat suppression, short-tau inversion recovery; inversion time, 250 ms; NSA, 2; b value, 0 and 1000 s/mm^2^; FOV, 240 × 240 mm; matrix, 96 × 125; slice thickness, 4 mm; number of slices, 22; acceleration factor, 2; and acquisition time, 3 min 30 s. Contrast-enhanced 3D-T1-weighted imaging with a gradient-echo sequence: slice orientation, sagittal; TR/TE, 5.3/2.4 ms; flip angle (FA), 10; fat suppression, spectral-attenuated inversion recovery; FOV, 250 × 225 mm; matrix, 256 × 256; slice thickness, 1 mm; number of slices, 180; acceleration factor, 1.8; and acquisition time, 3 min 24 s. The contrast-enhanced 2D-T1-weighted imaging parameters were the same as the non-contrast parameters.

### pCASL MRI protocol

The pulse sequence parameters for 3D TSE pCASL were as follows: TR/TE, 6000/40 ms; FA, 90°; labeling duration, 1650 ms; post-label delay, 1800 ms; number of shots, 3; FOV, 240 × 240 mm; matrix, 80 × 80; slice thickness, 4 mm; number of slices, 22; acceleration factor, 2.5; and acquisition time, 5 min 36 s. The labeling plane was set parallel to the imaging volume and perpendicular to the common carotid artery.

TBF was calculated according to the following equation^9^:

$${\text{TBF}} = \frac{{6000 \cdot {\uplambda } \cdot \left( {{\text{SI}}_{{{\text{control}}}} - {\text{SI}}_{{{\text{label}}}} } \right) \cdot {\text{e}}^{{\frac{{{\text{PLD}}}}{{{\text{T}}_{{1,{\text{ blood}}}} }}}} }}{{2 \cdot {\upalpha } \cdot {\text{T}}_{{1,{\text{blood }}}} \cdot {\text{SI}}_{{\text{PD }}} \cdot \left( {1 - {\text{e}}^{{ - \frac{{\uptau }}{{{\text{T}}_{{1,{\text{blood}}}} }}}} } \right)}}{ }\;\;\left[ {{\text{mL/1}}00\;{\text{g/min}}} \right]$$

where λ is the blood/tumor-tissue water partition coefficient (1.0 g/mL), and SI_control_ and SI_label_ are the time-averaged signal intensities in the control and label images, respectively. T_1,blood_ is the longitudinal relaxation time of blood (1650 ms), α is the labeling efficiency (0.85), SI_PD_ is the signal intensity of a proton density-weighted image, and τ is the label duration (1650 ms). The value of λ was 1.0 mL/g. To calculate TBF, we used the same model and conditions as those used for calculating blood flow in the brain.

### Image analysis

Image analysis was performed by using a custom software application developed in MATLAB 2020a. The custom software displays the ADC map and the pCASL map for the same patient side by side on the monitor. A slice image of each map for display can be moved. Two board-certified neuroradiologists (F.T and R.K) reviewed all MRI sequences. First, we identified the tumors on T1-weighted images, T2-weighted images, and contrast-enhanced T1-weighted images. The ROIs were manually drawn around the tumor margin in the maximum diameters on the ADC map by using the software. The ROIs were within an entire solid part of a tumor as much as visually traced, avoiding areas of necrosis, cyst, or hemorrhage. Then, the segmented ROI was copied from the ADC map and pasted to the pCASL image by using the software. The histogram features for each image were determined using those histograms. The following 10 objective features were determined as histogram features in the custom software: (1) minimum (min), (2) mean, (3) maximum (max), (4) 10th percentile, (5) 25th percentile, (6) 50th percentile, (7) 75th percentile, (8) 90th percentile, (9) skewness, and (10) kurtosis. The histogram features of TBF and ADC were measured twice in each ROI, and these measurements were averaged.

### Statistical analysis

Statistical analysis was performed by using SPSS v. 25.0 software (IBM SPSS Statistics for Windows, IBM Corp., Armonk, NY). Pearson’s chi-square test was utilized to assess comparison of sex, tumor sub-site, and diagnostic method among the tumor types, and one-way analysis of variance was utilized to assess comparison of age and tumor diameter among the tumor types. All 10 parameters of the TBF and ADC values were assessed. Significant differences among the groups were analyzed using one-way analysis of variance followed by Tukey post-hoc tests, after Shapiro–Wilk test, which was performed to assess the normality of data distribution. A *p* value of < 0.05 was considered to be indicative of statistical significance.

ROC curve analyses were performed to investigate the diagnostic performance of each parameter of TBF and ADC. All TBF parameters combined using binominal logistic regression were indicated as TBF_all_; all ADC parameters combined using binominal logistic regression were indicated as ADC_all_; and all TBF and ADC parameters combined using binominal logistic regression were indicated as TBF_all_ + ADC_all_. These terms were used in differentiating MTs from PAs, MTs from WTs, PAs from WTs, and MTs from BTs, including PAs and WTs. We considered AUC values < 0.7, 0.7–0.9, and > 0.9 to indicate low, medium, and high diagnostic performance, respectively. Cutoff values were calculated with the maximum of the Youden index (Youden index = sensitivity + specificity − 1). A *p* value of < 0.05 was considered significant to be indicative of statistical significance.

Interobserver agreement on TBF and ADC values between two readers was evaluated by ICC. ICCs are considered excellent if > 0.74^16^.

### Ethics statement

This study was approved by the ethics committee of Mie University School of Medicine, and the requirement for written informed consent was waived because of the retrospective study design. All study procedures were conducted according to the principles of the World Medical Association Declaration of Helsinki.

## Supplementary Information

Supplementary Figure S1.

Supplementary Tables.

## Data Availability

The datasets generated and analyzed during the current study are available from the corresponding author on reasonable request.
