# Author Correction: Tumor blood flow and apparent diffusion coefficient histogram analysis for differentiating malignant salivary tumors from pleomorphic adenomas and Warthin’s tumors

**DOI:** 10.1038/s41598-022-17426-2

**Published:** 2022-07-28

**Authors:** Fumine Tanaka, Maki Umino, Masayuki Maeda, Ryohei Nakayama, Katsuhiro Inoue, Ryota Kogue, Makoto Obara, Hajime Sakuma

**Affiliations:** 1 Department of Radiology, Mie University School of Medicine, 2-174 Edobashi, Tsu, Mie Japan; 2 Department of Neuroradiology, Mie University School of Medicine, 2-174 Edobashi, Tsu, Mie Japan; 3 Department of Electronic and Computer Engineering, Ritsumeikan University, 1-1-1 Noji-higashi, Kusatsu, Shiga Japan; 4 Department of Radiology, Mie University Hospital, 2-174 Edobashi, Tsu, Mie Japan; 5 MR Clinical Science, Philips Japan, 2-13-37 Konan, Minato, Tokyo, Japan

Correction to: *Scientific Reports* https://doi.org/10.1038/s41598-022-09968-2, published online 08 April 2022

The original version of this Article contained errors.

In the legends of Figures 1, 2 and 3,

“The region of interest (ROI) was manually drawn on the apparent diffusion coefficient (ADC) map of the software (**d**, *yellow*), and the ROI was copied from the ADC map to the TBF map of the software (**e**, *yellow*).”

now reads:

“The region of interest (ROI) was manually drawn on the apparent diffusion coefficient (ADC) map of the software (**e,** *yellow*), and the ROI was copied from the ADC map to the TBF map of the software (**d**, *yellow*).”

Additionally, the Author contributions statement,

“Study concept and design: T.T., M.U., and M.M. Acquisition of data: K.I. and M.O., development of the software for image analysis: R.N. Analysis and interpretation of data: F.T. and M.M. Drafting of the manuscript: F.T. and M.M. Statistical analysis: F.T. Study supervision: M.M. and H.S. All authors reviewed the manuscript.”

now reads:

“Study concept and design: F.T., M.U., and M.M. Acquisition of data: K.I. and M.O., development of the software for image analysis: R.N. Analysis and interpretation of data: F.T. and M.M. Drafting of the manuscript: F.T. and M.M. Statistical analysis: F.T. Study supervision: M.M. and H.S. All authors reviewed the manuscript.”

The original Article has been corrected.

