# Supplementary figures and images for: Tumor blood flow and apparent diffusion coefficient histogram analysis for differentiating malignant salivary tumors from pleomorphic adenomas and Warthin’s tumors
Source: Sci Rep. 2022 Apr 8;12:5947. doi: 10.1038/s41598-022-09968-2 (PMC8993800; doi:10.1038/s41598-022-09968-2)

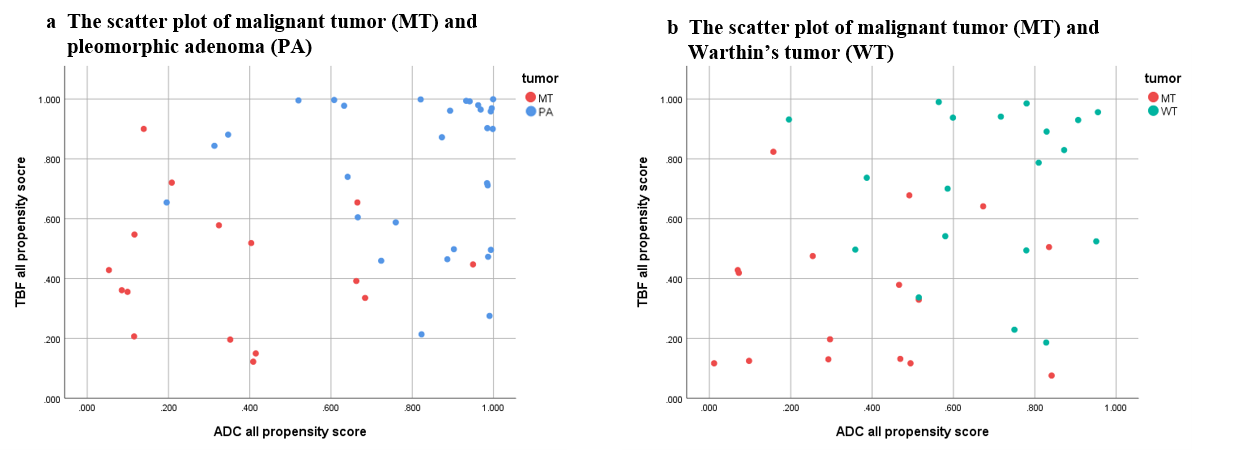

Supplement: Supplementary file 1 — Supplementary Figure S1. [file 41598_2022_9968_MOESM1_ESM.png]
