# Supplementary material for: Tumor blood flow and apparent diffusion coefficient histogram analysis for differentiating malignant salivary tumors from pleomorphic adenomas and Warthin’s tumors
Source: Sci Rep. 2022 Apr 8;12:5947. doi: 10.1038/s41598-022-09968-2 (PMC8993800; doi:10.1038/s41598-022-09968-2)
Supplement: Supplementary file 2 — Supplementary Tables. [file 41598_2022_9968_MOESM2_ESM.docx]

**Supplementary information**

**Table S1.** Shapiro–Wilk test for each parameter

| **Parameter** | **Tumor** | **Statistic** | ***p* value** |
| --- | --- | --- | --- |
| TBFmax | MT | 0.959 | 0.642 |
|  | WT | 0.938 | 0.241 |
|  | PA | 0.969 | 0.517 |
| TBFmin | MT | 0.768 | 0.001^*^ |
|  | WT | 0.933 | 0.201 |
|  | PA | 0.568 | 0.000* |
| TBFmean | MT | 0.929 | 0.234 |
|  | WT | 0.962 | 0.615 |
|  | PA | 0.870 | 0.002* |
| TBFmedian | MT | 0.935 | 0.293 |
|  | WT | 0.967 | 0.725 |
|  | PA | 0.872 | 0.002* |
| TBFpercentile 10% | MT | 0.868 | 0.026* |
|  | WT | 0.935 | 0.214 |
|  | PA | 0.746 | 0.000* |
| TBFpercentile 25% | MT | 0.922 | 0.179 |
|  | WT | 0.953 | 0.451 |
|  | PA | 0.825 | 0.000* |
| TBFpercentile 50% | MT | 0.935 | 0.293 |
|  | WT | 0.967 | 0.725 |
|  | PA | 0.872 | 0.002* |
| TBFpercentile 75% | MT | 0.912 | 0.128 |
|  | WT | 0.971 | 0.796 |
|  | PA | 0.906 | 0.011* |
| TBFpercentile 90% | MT | 0.941 | 0.356 |
|  | WT | 0.957 | 0.517 |
|  | PA | 0.924 | 0.034* |
| TBFskewness | MT | 0.907 | 0.103 |
|  | WT | 0.848 | 0.006* |
|  | PA | 0.965 | 0.417 |
| TBFkurtosis | MT | 0.889 | 0.054 |
|  | WT | 0.436 | 0.000* |
|  | PA | 0.910 | 0.015* |
| ADCmax | MT | 0.878 | 0.036* |
|  | WT | 0.835 | 0.004* |
|  | PA | 0.956 | 0.239 |
| ADCmin | MT | 0.956 | 0.593 |
|  | WT | 0.980 | 0.947 |
|  | PA | 0.989 | 0.984 |
| ADCmean | MT | 0.875 | 0.033* |
|  | WT | 0.831 | 0.003* |
|  | PA | 0.965 | 0.404 |
| ADCmedian | MT | 0.855 | 0.016* |
|  | WT | 0.851 | 0.007* |
|  | PA | 0.965 | 0.403 |
| ADCpercentile 10% | MT | 0.934 | 0.279 |
|  | WT | 0.867 | 0.013* |
|  | PA | 0.978 | 0.773 |
| ADCpercentile 25% | MT | 0.879 | 0.038* |
|  | WT | 0.864 | 0.011* |
|  | PA | 0.975 | 0.680 |
| ADCpercentile 50% | MT | 0.855 | 0.016* |
|  | WT | 0.851 | 0.007* |
|  | PA | 0.965 | 0.403 |
| ADCpercentile 75% | MT | 0.877 | 0.035* |
|  | WT | 0.830 | 0.003* |
|  | PA | 0.949 | 0.159 |
| ADCpercentile 90% | MT | 0.891 | 0.057 |
|  | WT | 0.791 | 0.001* |
|  | PA | 0.942 | 0.102 |
| ADCskewness | MT | 0.906 | 0.099 |
|  | WT | 0.986 | 0.987 |
|  | PA | 0.979 | 0.790 |
| ADCkurtosis | MT | 0.794 | 0.002* |
|  | WT | 0.962 | 0.620 |
|  | PA | 0.838 | 0.000* |

Abbreviations; TBF, tumor blood flow; ADC, apparent diffusion coefficient; max, maximum; min, minimum; MT, malignant tumor; WT, Warthin’s tumor; PA, pleomorphic adenoma

* *P*-value < 0.05

**Table S2. Receiver operating characteristic curve analysis of the parameters for differentiating MT from PA**

| **Parameters** | **AUC** | **Cutoff value** | **Sensitivity (%)** | **Specificity (%)** | **Youden index** |
| --- | --- | --- | --- | --- | --- |
| TBF max | 0.665 | 99.80 | 50.0 | 86.7 | 0.367 |
| TBF min | 0.731 | 6.89 | 56.3 | 83.3 | 0.396 |
| TBF mean | 0.740 | 66.82 | 50.0 | 93.3 | 0.433 |
| TBF 10th percentile | 0.738 | 6.26 | 93.8 | 46.7 | 0.404 |
| TBF 25th percentile | 0.742 | 13.48 | 93.8 | 53.3 | 0.471 |
| TBF 50th percentile | 0.744 | 20.06 | 93.8 | 50.0 | 0.438 |
| TBF 75th percentile | 0.740 | 77.91 | 50.0 | 93.3 | 0.433 |
| TBF 90th percentile | 0.721 | 83.98 | 50.0 | 93.3 | 0.433 |
| TBF skewness | 0.710 | 0.71 | 46.7 | 100.0 | 0.467 |
| TBF kurtosis | 0.798 | 0.00 | 66.7 | 93.8 | 0.604 |
| ADC max | 0.877 | 1.73 | 76.7 | 93.8 | 0.704 |
| ADC min | 0.856 | 1.01 | 70.0 | 93.8 | 0.638 |
| ADC mean | 0.879 | 1.30 | 80.0 | 87.5 | 0.675 |
| ADC 10th percentile | 0.885 | 1.15 | 73.3 | 93.8 | 0.671 |
| ADC 25th percentile | 0.885 | 1.26 | 73.3 | 93.8 | 0.671 |
| ADC 50th percentile | 0.867 | 1.38 | 73.3 | 93.8 | 0.671 |
| ADC 75th percentile | 0.883 | 1.38 | 80.0 | 87.5 | 0.675 |
| ADC 90th percentile | 0.873 | 1.48 | 80.0 | 87.5 | 0.675 |
| ADC skewness | 0.592 | 0.17 | 43.3 | 81.3 | 0.246 |
| ADC kurtosis | 0.598 | −0.15 | 68.8 | 53.3 | 0.221 |

Abbreviations; TBF, tumor blood flow (mL/100 g/min); ADC, apparent diffusion coefficient (×10^−3^ mm^2^/sec); max, maximum; min, minimum; MT, malignant tumor; PA, pleomorphic adenoma

**Table S3. Receiver operating characteristic curve analysis of the parameters for differentiating MT from WT**

| **Parameters** | **AUC** | **Cutoff value** | **Sensitivity (%)** | **Specificity (%)** | **Youden index** |
| --- | --- | --- | --- | --- | --- |
| TBF max | 0.829 | 151.61 | 73.7 | 87.5 | 0.612 |
| TBF min | 0.813 | 30.21 | 73.7 | 75.0 | 0.487 |
| TBF mean | 0.842 | 102.51 | 63.2 | 93.8 | 0.569 |
| TBF 10th percentile | 0.845 | 20.97 | 100.0 | 56.3 | 0.563 |
| TBF 25th percentile | 0.836 | 75.10 | 68.4 | 87.5 | 0.559 |
| TBF 50th percentile | 0.855 | 78.02 | 84.2 | 75.0 | 0.592 |
| TBF 75th percentile | 0.836 | 111.49 | 68.4 | 87.5 | 0.559 |
| TBF 90th percentile | 0.832 | 124.57 | 73.7 | 87.5 | 0.612 |
| TBF skewness | 0.651 | 0.34 | 43.8 | 89.5 | 0.332 |
| TBF kurtosis | 0.556 | 0.11 | 31.6 | 93.8 | 0.253 |
| ADC max | 0.671 | 1.15 | 93.8 | 57.9 | 0.516 |
| ADC min | 0.814 | 0.62 | 81.3 | 78.9 | 0.602 |
| ADC mean | 0.743 | 0.85 | 81.3 | 68.4 | 0.497 |
| ADC 10th percentile | 0.806 | 0.80 | 62.5 | 89.5 | 0.520 |
| ADC 25th percentile | 0.783 | 0.76 | 75.0 | 73.7 | 0.487 |
| ADC 50th percentile | 0.763 | 0.80 | 87.5 | 63.2 | 0.507 |
| ADC 75th percentile | 0.730 | 0.91 | 87.5 | 63.2 | 0.507 |
| ADC 90th percentile | 0.727 | 1.02 | 87.5 | 68.4 | 0.559 |
| ADC skewness | 0.701 | 0.08 | 68.4 | 75.0 | 0.434 |
| ADC kurtosis | 0.592 | 0.13 | 63.2 | 62.5 | 0.257 |

Abbreviations; TBF, tumor blood flow (mL/100 g/min); ADC, apparent diffusion coefficient (×10^−3^ mm^2^/sec); max, maximum; min, minimum; MT, malignant tumor; WT, Warthin’s tumor

**Table S4. Receiver operating characteristic curve analysis of the parameters for differentiating PA from WT**

| **Parameters** | **AUC** | **Cutoff value** | **Sensitivity (%)** | **Specificity (%)** | **Youden index** |
| --- | --- | --- | --- | --- | --- |
| TBF max | 0.954 | 85.41 | 100.0 | 76.7 | 0.767 |
| TBF min | 0.944 | 6.67 | 94.7 | 83.3 | 0.781 |
| TBF mean | 0.954 | 68.30 | 84.2 | 93.3 | 0.775 |
| TBF 10th percentile | 0.946 | 20.28 | 100.0 | 76.7 | 0.767 |
| TBF 25th percentile | 0.946 | 27.39 | 100.0 | 73.3 | 0.733 |
| TBF 50th percentile | 0.951 | 71.21 | 84.2 | 93.3 | 0.775 |
| TBF 75th percentile | 0.963 | 70.65 | 89.5 | 90.0 | 0.795 |
| TBF 90th percentile | 0.968 | 82.30 | 89.5 | 93.3 | 0.828 |
| TBF skewness | 0.821 | 0.27 | 70.0 | 89.5 | 0.595 |
| TBF kurtosis | 0.695 | −0.30 | 80.0 | 63.2 | 0.432 |
| ADC max | 0.870 | 1.27 | 100.0 | 68.4 | 0.684 |
| ADC min | 0.960 | 0.76 | 93.3 | 94.7 | 0.881 |
| ADC mean | 0.960 | 1.04 | 96.7 | 84.2 | 0.809 |
| ADC 10th percentile | 0.984 | 0.79 | 100.0 | 89.5 | 0.895 |
| ADC 25th percentile | 0.979 | 0.95 | 96.7 | 89.5 | 0.861 |
| ADC 50th percentile | 0.962 | 0.97 | 100.0 | 78.9 | 0.789 |
| ADC 75th percentile | 0.933 | 1.15 | 93.3 | 84.2 | 0.775 |
| ADC 90th percentile | 0.898 | 1.20 | 96.7 | 78.9 | 0.756 |
| ADC skewness | 0.635 | 0.41 | 47.4 | 76.7 | 0.240 |
| ADC kurtosis | 0.674 | 0.06 | 73.7 | 66.7 | 0.404 |

Abbreviations; AUC, area under the curve; TBF, tumor blood flow (mL/100 g/min); max, maximum; min, minimum; ADC, apparent diffusion coefficient (×10^−3^ mm^2^/sec); PA, pleomorphic adenoma; WT, Warthin’s tumor

**Table S5. Interobserver agreement**

| **Parameters** | **ICC** |
| --- | --- |
| TBF max | 0.966 |
| TBF min | 0.827 |
| TBF mean | 0.995 |
| TBF 10th percentile | 0.919 |
| TBF 25th percentile | 0.962 |
| TBF 50th percentile | 0.997 |
| TBF 75th percentile | 0.998 |
| TBF 90th percentile | 0.991 |
| TBF skewness | 0.957 |
| TBF kurtosis | 0.930 |
| ADC max | 0.918 |
| ADC min | 0.983 |
| ADC mean | 0.997 |
| ADC 10th percentile | 0.991 |
| ADC 25th percentile | 0.999 |
| ADC 50th percentile | 0.995 |
| ADC 75th percentile | 0.993 |
| ADC 90th percentile | 0.989 |
| ADC skewness | 0.713 |
| ADC kurtosis | 0.892 |

Abbreviations; ICC, intraclass correlation coefficient; TBF, tumor blood flow; max, maximum; min, minimum; ADC, apparent diffusion coefficient
